# A differentiation protocol for generating pancreatic delta cells from human pluripotent stem cells

**DOI:** 10.3389/fcell.2024.1490040

**Published:** 2024-10-18

**Authors:** Tongran Zhang, Nannan Wang, Zhiying Liao, Jingyi Chen, Hao Meng, Haopeng Lin, Tao Xu, Lihua Chen, Ling-Qiang Zhu, Huisheng Liu

**Affiliations:** ^1^ Department of Pathophysiology, School of Basic Medicine, Tongji Medical College, Huazhong University of Science and Technology, Wuhan, Hubei, China; ^2^ Department of Testing and Diagnosis Technology Research, Guangzhou National Laboratory, Guangzhou, Guangdong, China; ^3^ College of Life Science and Technology, Huazhong University of Science and Technology, Wuhan, Hubei, China; ^4^ School of Biomedical Engineering, Guangzhou Medical University, Guangzhou, Guangdong, China; ^5^ School of Biomedical Sciences and Engineering, South China University of Technology, Guangzhou International Campus, Guangzhou, Guangdong, China

**Keywords:** hPSCs, stem cell differentiation, pancreatic delta cells, FGF, islet organoids

## Abstract

In this protocol, we detail a seven-stage differentiation methodology for generating pancreatic delta cells (SC-delta cells) from human pluripotent stem cells (hPSCs). In the first step, definitive endoderm is generated by activin A and CHIR99021, followed by induction of primitive gut tube and posterior foregut by treatment with FGF7, SANT1, LDN193189, PdBU, and retinoic acid (RA). The subsequent endocrine generation and directed SC-delta cell induction is achieved by a combined treatment of the FGF7 with FGF2 during stage 4 and 5, together with RA, XXI, ALK5 inhibitor II, SANT1, Betacellulin and LDN193189. The planar cultivation is converted to a suspended system after stage 5, allowing cells to aggregate into delta cell-containing spheroids. The differentiation takes approximately 4-5 weeks for delta cell generation and an additional 1-2 weeks for cell expansion and evaluation. We believe that this amenable and simplified protocol can provide a stable source of SC-delta cells from efficient differentiation, facilitating further investigation of the physiological role of delta cells as well as refinement of islet cell therapeutic strategies.

## 1 Introduction

Diabetes mellitus is a chronic and prevalent disease with serious complications such as hyperglycemia, diabetic nephropathy, diabetic ophthalmopathy, neuropathy, and cardiovascular disease, which has caused a huge social burden worldwide. Human pancreatic islets are composed of endocrine and exocrine cells (Olaniru et al., 2023). The three main types of endocrine cells: alpha cells, beta cells and delta cells can secret different hormones to finely regulate glucose homeostasis and energy metabolism (Svendsen and Holst, 2021; Huang et al., 2024; Raskin and Unger, 1978). Specifically, when blood glucose elevates, beta cells release insulin to facilitate the utilization of glucose, whereas when blood glucose is below the physiological level, alpha cells increase hepatic glucose output by releasing glucagon, resulting in blood glucose return (Jiang and Zhang, 2003; Omar-Hmeadi et al., 2020). Not only the dysfunction of beta cells (Eizirik et al., 2020; Hudish et al., 2019) and alpha cells (Del Prato and Marchetti, 2004), but also the dysfunction of delta cells (Lawlor et al., 2017) will lead to impaired metabolic balance and significantly increase the risk of diabetes mellitus.

Although delta cells make up a relatively small proportion of pancreatic islets, they play important roles in maintaining blood glucose set point within a narrow physiological range by effectively inhibiting the release of insulin and glucagon through somatostatin receptor (SSTR) on both alpha and beta cells (Strowski et al., 2000; Abdel-Halim et al., 1993; Karimian et al., 2013; Kailey et al., 2012). There are pieces of evidence showing that the dysfunction of delta cells is related to development of diabetes mellitus. For example, delta cell death as well as impaired somatostatin (SST) secretion were found in animal models of diabetes mellitus (Collins et al., 2008; Guardado Mendoza et al., 2015). Moreover, the reduced SSTR2 expression detected in type 2 diabetes suggested the SST resistance (Omar-Hmeadi et al., 2020). On the other hand, SST infusion and usage of its analog can improve glucose homeostasis (Di Mauro et al., 2001; Gerich et al., 1977). Consequently, research into delta cells is gaining increasing attention, and the regulation of delta cell function is emerging as a promising new avenue for the treatment of diabetes (Yue et al., 2013). Because of their essential roles in keeping the normal physiological function of pancreatic islets, delta cells are indispensable in the construction of physiological pancreatic islets.

However, current pathophysiological studies on pancreatic delta cells are greatly constrained by the shortage of cell resources. Although some laboratories have reported the generation of a small number of SST-positive cells upon differentiation of alpha or beta cells from human pluripotent stem cells, the overall percentage of these cells is extremely low (Rezania et al., 2014; Veres et al., 2019; Hogrebe et al., 2020; Loretelli et al., 2020; Peterson et al., 2020). Moreover, the characteristics and functions of these cells remain largely uncharacterized (Veres et al., 2019; Hogrebe et al., 2020). Thus, the missing of delta cells results in a large discrepancy between islet-organoids and physiologic pancreatic islets, impeding the exploration of the physiological roles of delta cells.

With the development of stem cell biology and regenerative medicine (Du et al., 2022), hPSC-derived functional pancreatic beta cells and alpha cells (Rezania et al., 2014; Veres et al., 2019; Hogrebe et al., 2020; Loretelli et al., 2020; Peterson et al., 2020) have been demonstrated to have the potential to reverse diabetes in numerous preclinical and clinical trials (Hering et al., 2023; Marfil-Garza et al., 2022) and becoming increasingly well-developed (Peterson et al., 2020; Ma et al., 2022; Liang et al., 2023). Nonetheless, the hPSC-derived islet transplantation remains deficient currently, as the methods for differentiation of delta cells have not been established. It is the absence of delta cells that led to significant differences in cellular composition and spatial structure of the islet-like organoids versus physiologic islets (Arrojo et al., 2019), thus limiting their clinical researches and applications. In conclusion, obtaining a stable and sufficient supply of delta cells is highly desirable.

### 1.1 Development of the protocol

Developmental biology studies have revealed that pancreatic delta cells and beta cells share very similar developmental and signaling pathways. Both cell types originate from PDX1-, NGN3-, and NEUROD1-positive islet progenitors and subsequently express PAX4, NKX2.2, and PAX6 during the formation of endocrine progenitor cells (Chiang and Melton, 2003; Gu et al., 2002; Sosa-Pineda et al., 1997). Later, the delta cells specifically express the transcription factor HHEX and restrict the expression of NKX6.1 (Oster et al., 1998; Zhang et al., 2014). Based on these similarities, our strategy is to screen for small molecules that can specifically induce delta cell differentiation based upon the current beta cell differentiation method, thus to establish a method for the directed differentiation of pancreatic delta cells.

We performed a low-throughput screening of small molecular compounds/fibroblast growth factors (Ameri et al., 2010; Ye et al., 2005; Elghazi et al., 2002) in the established islet organoid differentiation system (Rezania et al., 2014) and determined the expression of various cell marker genes by qRT-PCR. We found that during primitive gut tube (S2) and posterior foregut (S3) induction, FGF7 with a concentration of 50 ng/mL could increase the expression of PDX1 and CHGA, the markers of pancreatic endoderm/endocrine precursors (Afelik et al., 2006). Notably during the following pancreatic endoderm (S4) and pancreatic endocrine precursor cell induction (S5), FGF2 with a concentration of 20 ng/mL was shown to significantly promote the expression of delta cell marker gene SST and the specific transcription factor HHEX, as well as their protein expression during endocrine cell induction (S6). Based upon this, we further explored the concentration and combination of FGF2 with FGF7 (FGF2/7), using qRT-PCR, Western blot, flow cytometric analysis, and immunocytochemistry (ICC) experiments. Although there was no significant difference between FGF7 and FGF2/7 combination during early stages, the combination of 20 ng/mL FGF2 with 50 ng/mL FGF7 during S4 and S5 was able to synergically increase the proportion of SST-positive delta cells, while decreasing alpha and beta cells at the end stage of differentiation, thus confirming the efficiency and robustness of our delta cell differentiation method (Chen et al., 2024).

### 1.2 Applications of the method

The efficient differentiation protocol of human pancreatic delta cells can provide a stable source of delta cells, which will greatly facilitate the study of the physiological role of delta cells in the development of diabetes and provide systematic support for screening of delta cell targeting drugs. Moreover, the study of physiologically mature islet organoids containing delta cells will promote the development and refinement of cell therapeutic strategies and provide guidance for pre-clinical studies and clinical applications.

### 1.3 Comparison with other methods

In contrast to established protocols for differentiating pluripotent stem cells into pancreatic alpha and beta cells, which report a fraction of delta cells as a by-product, our method significantly increases the proportion of pancreatic delta cells that are functional *in vivo* and *in vitro*. Here, our seven-step approach combining planar and suspension cultures provides stable and efficient production of delta cells with quality control to ensure differentiation efficiency and save differentiation costs.

## 2 Materials and reagents

### 2.1 Cell lines

In this SC-delta cell differentiation protocol, we used a source of hPSCs, including both embryonic stem cells (ESCs) and induced pluripotent stem cells (iPSCs).

• The H1 and H9 cell lines were obtained from WiCell Research Institute.
• The iPS cell line UE005 and UC017 were kind gifts from Dr. Guangjin Pan.

### 2.2 Stem cell culture

• mTeSR1 complete kit (Stemcell Technologies, cat. no. 85850)
• Matrigel (Corning, cat. no. 354277)
• Gentle cell dissociation reagent (Stem cell, cat. no. 7174)
• Y-27632 (Stemcell, cat. no. 72307)
• TrypLE Express (Thermo Fisher, cat. no. 12604039)
• Accutase (Stemcell Technologies, cat. no. 07920)
• DMEM/F12 (Thermo Fisher, cat. no. 11330032)

### 2.3 Basal medium components

• 500 mL MCDB 131 (Thermo Fisher, cat. no. 10372019)
• Glucose (Sigma, cat. no. G8769)
• Sodium bicarbonate (Sigma, cat. no. S6297)
• Bovine serum albumin (BSA; Proliant Biologicals, cat. no. 68700)
• GlutaMAX (Invitrogen, cat. no. 35050079)
• Penicillin/streptomycin (P/S) solution (Thermo Fisher, cat. no. 15070063)
• ITS-X ((Invitrogen, cat. no. 51500056)
• Ascorbic acid (Vitamin C; Sigma, cat. no. A4544)

### 2.4 Differentiation factors

• Activin A (StemCell Technologies, cat. no. 78001)
• CHIR99021 (Stemgent, cat. no. 04-0004-10)
• FGF7 (StemCell, cat. no. 78046)
• FGF2 (Med Chem Express, cat. no. HY-P7004)
• Retinoic acid (Sigma, cat. no. R2625)
• SANT-1 (Sigma, cat. no. S4572)
• LDN193189 (Reprocell, cat. no. 40074)
• PdBU (Millipore, cat. no. 524390)
• ALK5i II (Cell Guidance Systems, cat. no. SM09-50)
• XXI (Millipore, cat. no. 595790)
• Betacellulin (Med Chem Express, cat. no. HY-P7005)
• Heparin (Sigma, cat. no. H3149-500KU)
• N-acetyl cysteine (Sigma, cat. no. A9165-100G)
• R428 (Selleck, cat. no. S2841)
• α-Tocopherol (Sigma, cat. no. T3251)
• Zinc sulfate (Sigma, cat. no. Z0251)
• PBS for dissolving factors (HyClone, cat. no. SH30256.01)
• Dimethyl sulfoxide for dissolving factors (DMSO) (MilliporeSigma, cat. no. D4540-100ML)

### 2.5 Antibodies

• Rabbit anti-FOXA2 (Abcam, cat. no. ab108422; RRID: AB_11157157)
• Mouse anti-SOX17 (Abcam, cat. no. ab84990; RRID: AB_1861437)
• Goat anti-PDX1 (Abcam, cat. no. ab47383; RRID: AB_2162359)
• Mouse anti-NKX6.1 (Abcam, cat. no. ab268088)
• Rat anti-SST (for ICC, Abcam, cat. no. ab30788; RRID: AB_778010)
• Purified Mouse Anti-Human Somatostatin (for flow cytometry, BD, cat. no. 566031; RRID: AB_2739475)
• Rabbit anti-Chromogranin A (for flow cytometry, Abcam, cat. no. ab45179; RRID: AB_726879)
• Anti-rabbit alexa fluor 488 (Invitrogen, cat. no. A21206; RRID: AB_2535792)
• Anti-mouse alexa fluor 546 (Invitrogen, cat. no. A10036; RRID: AB_11180613)
• Anti-goat alexa fluor 488 (Invitrogen, cat. no. A11055; RRID: AB_2534102)
• Anti-rat alexa fluor 488 (Invitrogen, cat. no. A21208; RRID: AB_2535794)
• Anti-mouse alexa fluor 488 (Invitrogen, cat. no. A21202; RRID: AB_141607)
• Anti-goat alexa fluor 594 (Invitrogen, cat. no. A11058; RRID: AB_2534105)
• Anti-rabbit alexa fluor 647 (Abcam, cat. no. ab150075; RRID: AB_2752244)

### 2.6 Other reagents

• DAPI (Thermo Fisher Scientific, cat. no. 622492)
• 4% paraformaldehyde (PFA, Sangon Biotech, cat. No. E672002)
• Triton X-100 (Thermo Scientific, cat. no. 28314)
• Donkey serum (Solarbio, cat. no. SL050-100 mL)
• OCT solution (Biosharp, cat. no. BL557A)
• Perm/Wash buffer (BD, cat. no. 554723)
• RNeasy Mini Kit (Qiagen, cat. no. 74106)
• DNase kit (Qiagen, cat. no. 79254)
• RT reagent Kit with gDNA Eraser (TAKARA, cat. no. RR047A)
• SYBR Premix Ex TaqII (TAKARA, cat. no. RR820A)

### 2.7 Equipments

• Pipettors (Eppendorf, cat. no. 3120000216; 3120000232; 3120000259; 3120000267)
• Pipet-aid (Gilson, cat. no. F110120)
• Cell-counting system (Sigma-Aldrich, cat. no. Z359629-1EA)
• Orbital shaker (NEST, cat. no. 105008)
• Light microscope (RWD, cat. no. G2020123607)
• 37°C water bath (Bluepard, cat. no. DK-8D)
• Confocol microscope (Nikon A1Rsi microscope; Zeiss LSM800 confocal microscope)
• Flow cytometer (BD Biosciences, LSR-Fortessa)
• qRT-PCR thermocycler (Bio-Rad, cat. no. CFX96 Touch)
• Centrifuges (Cence, cat. no. L600-A; Eppendorf, cat. no. 5425R)
• −20°C freezers (Haier, cat. no. DW-25L262)
• −80°C freezers (Thermo Fisher, cat. no. 906-GP) freezers
• Humidified cell culture incubator (Thermo Fisher Scientific, cat. no. 51032719)
• Biosafety cabinet (Thermo Fisher Scientific, cat. no. 1389)

### 2.8 Consumables

• Serological pipettes (NEST, cat. no. 326001, 327001; Corning, cat. no. 4489)
• Sterile pipette tips (Kirgen, cat. no. KG1333, KG1232, KG1031)
• 1.5 mL microcentrifuge tubes (Axygen, cat. no. MCT-150-C)
• 50 mL conical tubes (Corning, cat. no. 430829)
• 24-well tissue culture treated plates (Corning, cat. no. 3524)
• 6-well tissue culture treated plates (Corning, cat. no. 3516)
• Ultra-low attachment 6-well plates (Corning, cat. no. 3471)
• Disposable sterile filter systems (Nalgene, cat. no. 566–0020)
• 96-well qRT-PCR reaction plates (Bio-Rad, cat. no. HSP9655)
• Tubes for flow cytometry (Falcon, cat. no. 352235)
• Slice box (Easybio, cat. no. BE6153)
• Micro coverslips (CITOTEST, cat. no. 10212450c)
• Glass slides (CITOTEST, cat. no. 188105)

### 2.9 Reagents preparation

#### 2.9.1 Matrigel solution

Dilute Matrigel using DMEM/F12 to final concentration of 0.12 mg Matrigel per mL of DMEM. Dilution factor is lot-dependent. Pre-cool pipette tips and operate on ice. Table 1 suggests the volumes of Matrigel coating and medium feeding.

**TABLE 1:** Coating and feeding volumes for different cell culture ware.

Culture ware	Growth area (cm^2^)	Volume of coating	Volume of feeding
24-well plate	1.9	0.3 mL	0.5 mL/well
6-well plate	9.5	0.7 mL	2 mL/well
6-well plate (for suspension)	9.5	-	3 mL/well

#### 2.9.2 mTeSR1 medium

Mix mTeSR1 5 × supplement (store at −20°C) with mTeSR1 basal medium (store at 4°C).

#### 2.9.3 Basal medium

Components of S1 to S7 basal media are detailed in Table 2. Use MCDB131 to prepare basal media and sterile filter. Store the basal medium at 4°C.

**TABLE 2:** Basal medium components.

Basal media	S1/S2	S3/S4	S5/S6	S7
Reagent	500 mL MCDB131	500 mL MCDB131	500 mL MCDB131	500 mL MCDB131
BSA	2.5 g	10 g	10 g	10 g
Basal	0.9 g Glucose	0.9 g Glucose	1.8 g Glucose	1.8 g Glucose
	0.75 g NaHCO_3_	1.25 g NaHCO_3_	0.875 g NaHCO_3_	0.875 g NaHCO_3_
	22 mg Vitamin C	22 mg Vitamin C	22 mg Vitamin C	5 mL Glutamax
	5 mL Glutamax	5 mL Glutamax	5 mL Glutamax	5 mL P/S
	5 mL P/S	5 mL P/S	5 mL P/S	2.5 mL ITS-X
	10 μL ITS-X	2.5 mL ITS-X	2.5 mL ITS-X	

#### 2.9.4 Differentiation factors

The final concentration and dilution factors from stock aliquots of differentiation factors are listed in Table 3. Dissolve differentiation factors to stock concentration using solvents suggested by manufacturers, and prepare into appropriate aliquots. Factors can be stored at −80°C for long time storage (up to 1 year) and −20°C in the short term. Add fresh factors to the basal media prior to each feed.

**TABLE 3:** Final concentration of differentiation factors.

	Day of differentiation	Basal medium	Factor	Stock concentration	Final concentration	Dilution from stock
Stage 0	S1D0	mTeSR1	Y-27362	10 mM	10 μM	1:1000
Stage 1	S1D1	S1 Basal	Activin A	100 μg/mL	100 ng/mL	1:1000
			CHIR99021	3 mM	3 μM	1:1000
	S1D2	S1 Basal	Activin A	100 μg/mL	100 ng/mL	1:1000
			CHIR99021	3 mM	0.3 μM	1:10000
	S1D3	S1 Basal	Activin A	100 μg/mL	100 ng/mL	1:1000
Stage 2	S2D1-S2D2	S2 Basal	FGF7	100 μg/mL	50 ng/mL	1:2000
Stage 3	S3D1-S3D2	S3 Basal	RA	10 mM	1 μM	1:10000
			SANT-1	10 mM	0.25 μM	1:40000
			LDN193189	5 mM	100 nM	1:50000
			PdBU	2 mM	500 nM	1:4000
			Y27632	10 mM	10 μM	1:1000
			FGF7	100 μg/mL	50 ng/mL	1:2000
Stage 4	S4D1-S4D3	S4 Basal	RA	10 mM	0.1 μM	1:100000
			SANT-1	10 mM	0.25 μM	1:40000
			LDN193189	5 mM	100 nM	1:50000
			PdBU	2 mM	100 nM	1:20000
			Y27632	10 mM	10 μM	1:1000
			ALK5i-II	100 mM	10 μM	1:10000
			FGF2	20 μg/mL	20 ng/mL	1:1000
			FGF7	100 μg/mL	50 ng/mL	1:2000
Stage 5	S5D1-S5D3	S5 Basal	RA	10 mM	0.05 μM	1:200000
			SANT-1	10 mM	0.25 μM	1:40000
			LDN193189	5 mM	100 nM	1:50000
			ZnSO_4_	100 mM	10 μM	1:10000
			ALK5i-II	100 mM	10 μM	1:10000
			XXI	10 mM	1 μM	1:10000
			Betacellulin	100 μg/mL	20 ng/mL	1:5000
			Heparin	10 mg/mL	10 μg/mL	1:1000
			FGF2	20 μg/mL	20 ng/mL	1:1000
			FGF7	100 μg/mL	50 ng/mL	1:2000
Stage 6	S6D1-S6D5	S6 Basal	LDN193189	5 mM	100 nM	1:50000
			ZnSO_4_	100 mM	10 μM	1:10000
			XXI	10 mM	0.1 μM	1:100000
			Heparin	10 mg/mL	10 μg/mL	1:1000
			NAC	500 mM	1 mM	1:500
			R428	10 mM	2 μM	1:5000
Stage 7	S7D1-S7D21	S7 Basal	Heparin	10 mg/mL	10 μg/mL	1:1000
			ZnSO_4_	100 mM	10 μM	1:10000
			NAC	500 mM	1 mM	1:500
			α-Tocopherol	10 mM	10 μM	1:1000

## 3 Methods

### 3.1 Experimental design

**FIGURE 1:**
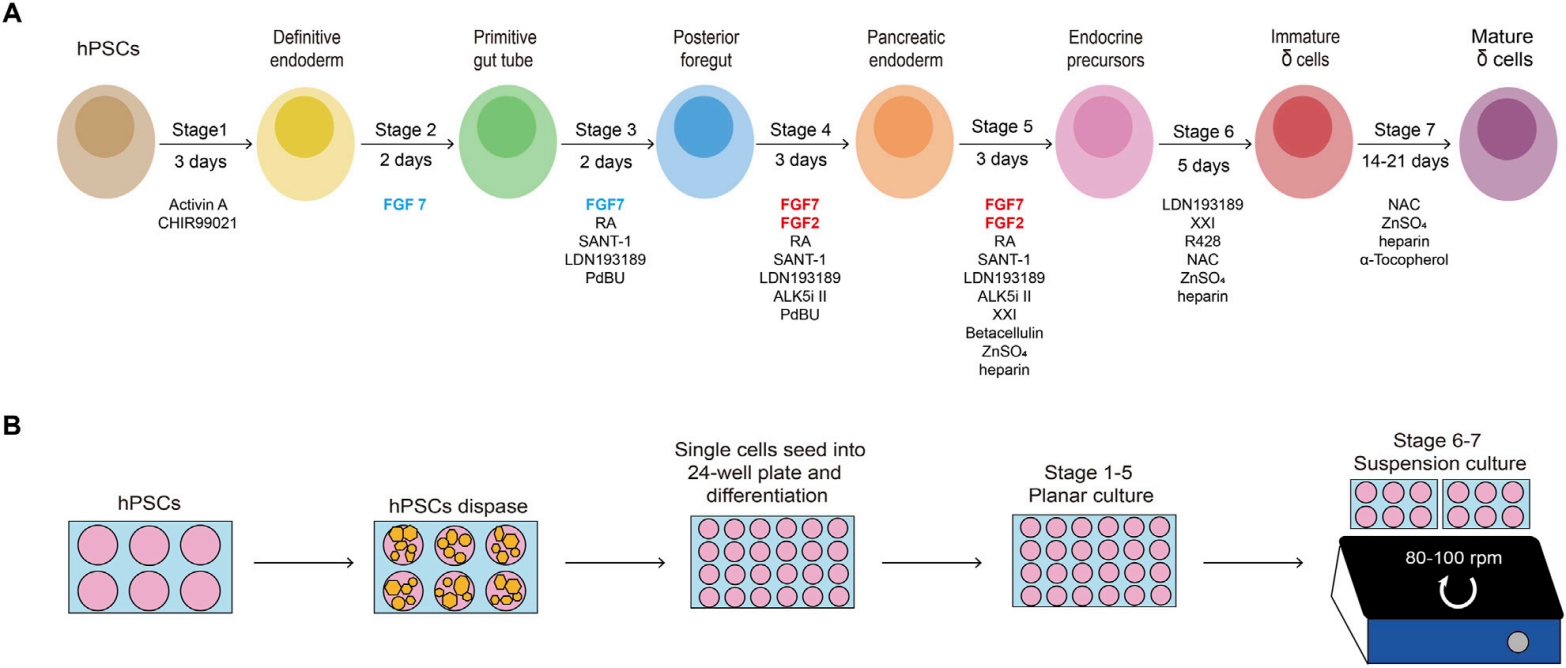
Schematic of the protocol and timeline. **(A)** Schematic of the seven-stage delta-cell differentiation protocol. hPSCs are directed differentiated into delta cells with seven progressive stages through several intermediate cell types, each of which is driven by specific growth factors and small molecules. **(B)** The hPSCs expansion in 6-well plates, planar culturing in 24-well plates (stage 1–5) and suspension culturing in Ultra-low attachment 6-well plates (stage 6-7).

Here, we describe in detail a stepwise differentiation protocol for directed generating functional pancreatic delta cells from hPSCs as shown in Figure 1. The methodology consists of seven stages to activate or inhibit various signaling pathways using small molecules and growth factors (Figure 1A), dedicating to recreate the organogenesis of pancreatic islets in culture medium. The methods to assess the cells are also detailed including quality control at critical checkpoints as well as determination of cell composition at the endpoint.

### 3.2 hPSC culture (stage 0, steps 1–13)

**FIGURE 2:**
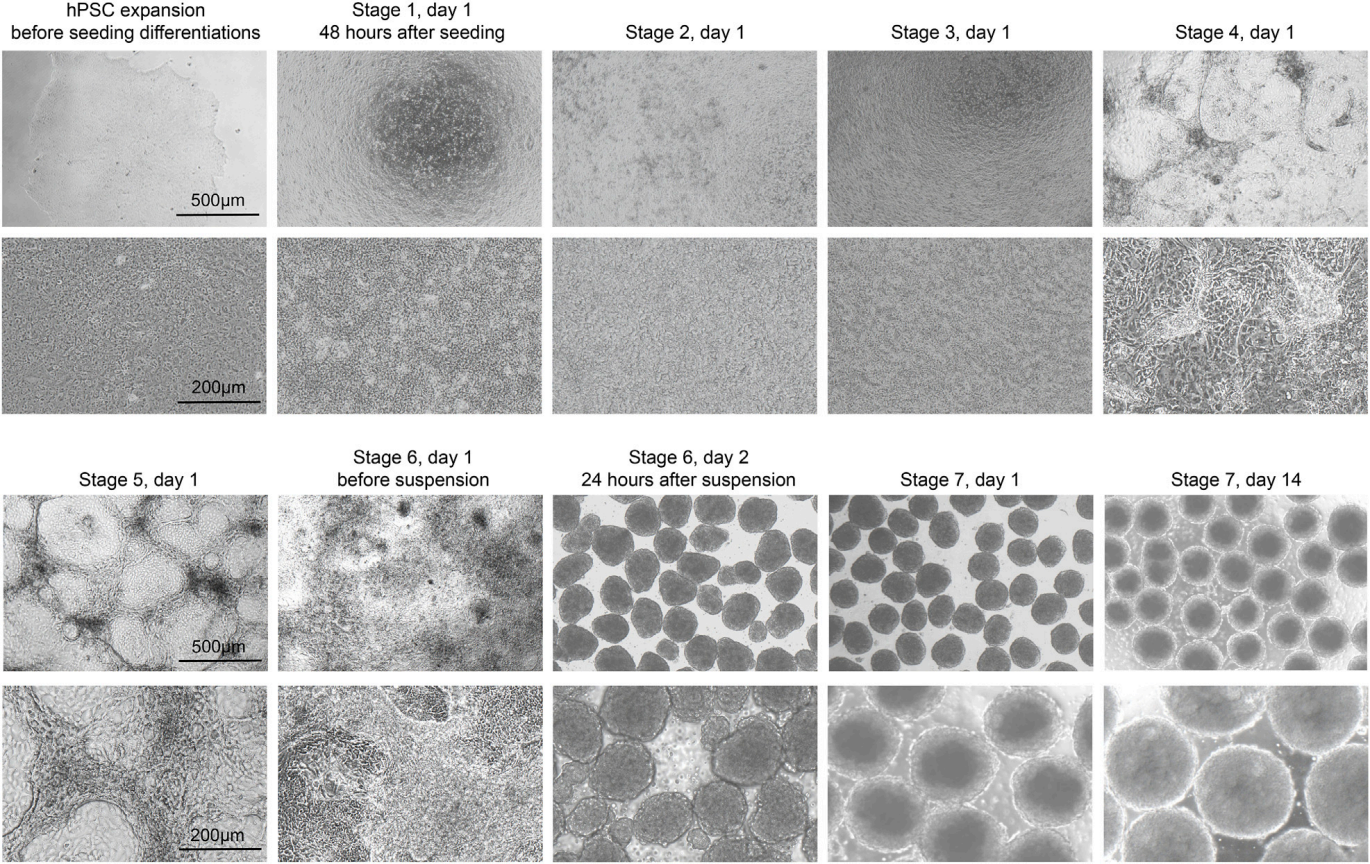
Morphology of differentiating cells. Representative brightfield images of the H1 cell line at the beginning of each stage of the protocol. Low magnification (top, scale bar, 500 µm) and high magnification (bottom, scale bar, 200 µm).

hPSCs are seeded onto Matrigel-coated 6-well plates at a density of 0.2 × 10^5^ cells/cm^2^ and cultured in mTeSR1 (Figure 2). When cells reach approximately 80% confluency after 6∼7 days, they are dispersed into a) single cells using TrypLE and seeded onto new Matrigel-coated 24-well plates at a density of 1.25 × 10^5^ cells/cm^2^ for differentiation; or b) small cell colonies using gentle cell dissociation reagent and seeded onto new Matrigel-coated plates with mTeSR1 with 10 μM Y-27632 (Rho-kinase inhibitor) for passage.

### 3.3 Definitive endoderm (stage 1, steps 14–17)

**FIGURE 3:**
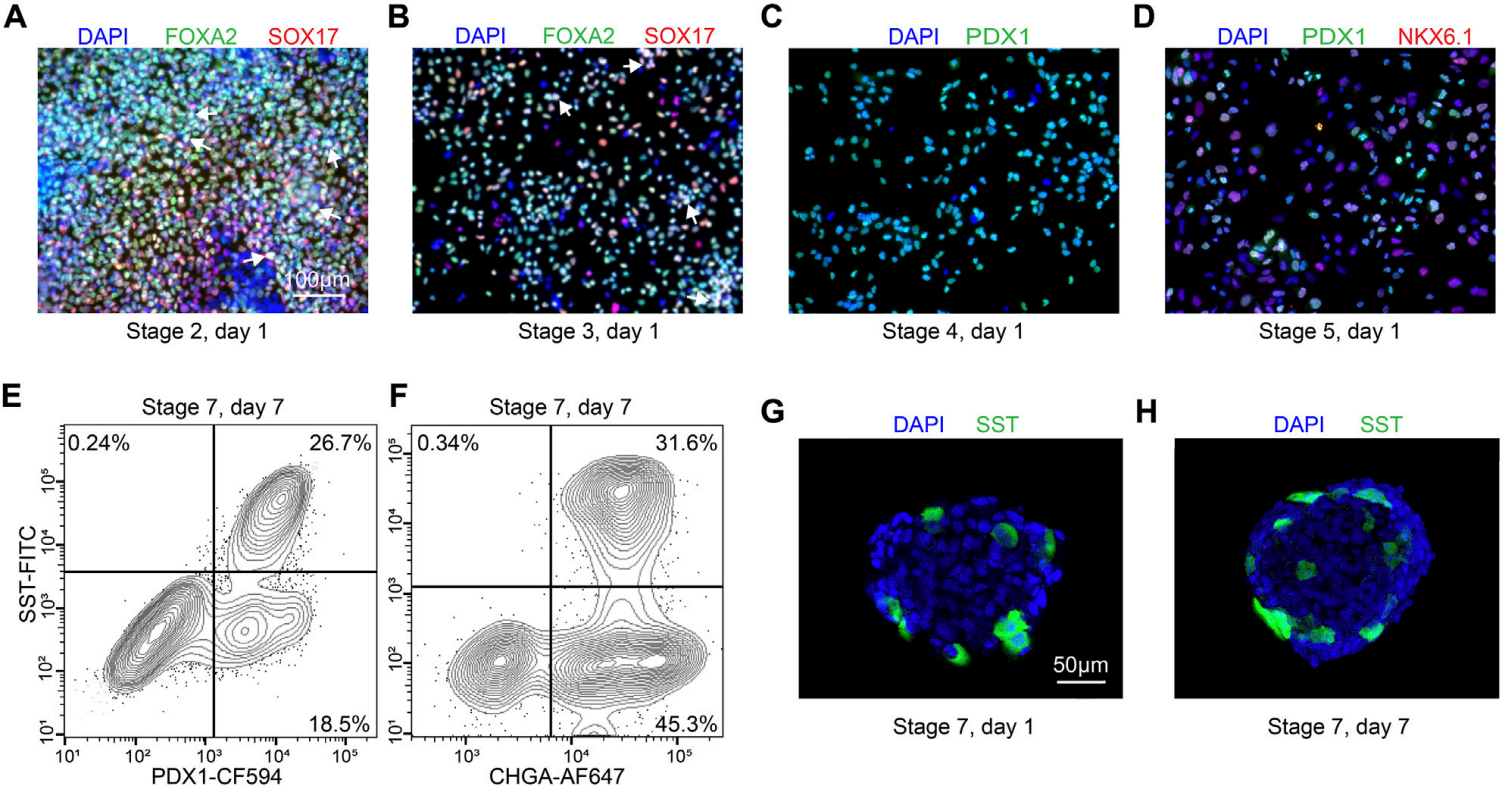
Quality control during differentiation and expected outcomes **(A–D)** ICC of key markers to quantitatively assess the quality of differentiation for H1 cell line at S2D1, S3D1, S4D1 and S5D1. **(A)** FOXA2 (green), SOX17 (red), DAPI (blue). White arrows point out typical FOXA2+/SOX17+ cells. **(B)** FOXA2 (green), SOX17 (red), DAPI (blue). White arrows point out typical FOXA2+/SOX17+ cells. **(C)** PDX1 (green), DAPI (blue). **(D)** PDX1 (green), NKX6.1 (red), DAPI (blue). **(E, F)** Flow cytometry to access the efficiency of delta cell generation at stage 7. **(G, H)** Immunostaining of histological sections of stage 7 cell clusters for quantitatively assessing delta cell marker SST (green). Scale bars, 100 µm **(A–D)** and 50 µm **(G–H)**.

The stem cells should be confluent 48 h after seeding onto 24-well plates, and mTeSR1 is replaced with stage 1 day 1 (S1D1) medium containing Activin A (TGF-β superfamily member) and CHIR99021 (Wnt agonist). 24 h later, the medium is replaced with S2D2 medium containing Activin A and a lower concentration of CHIR99021. The subsequent S1D3 medium used contains only Activin A. At the end of this stage, there should be more than 90% of the cells expressing the endoderm markers FOXA2 and SOX17 (Figure 3A). As a quality control checkpoint, high expression of these markers is critical to the success of the protocol, so it is important to optimize this step in each cell line prior to subsequent differentiation stages. All the markers for quality control can be checked by ICC visually or flow cytometry quantitatively.

### 3.4 Primitive gut tube (stage 2, steps 18–20)

During stage 2, the definitive endoderm cells are driven towards primitive gut tube with the keratinocyte growth factor (FGF7) treatment. At the end of S2D2, FOXA2 and SOX17 as reliable markers should be co-expressed in >85% of cells (Figure 3B) to distinguish the differentiation efficiency into SC-delta cells.

### 3.5 Posterior foregut (stage 3, steps 21–23)

In the 2 days of stage 3, the cells are converted to posterior foregut with FGF7 accompanied by a high concentration of Retinoic Acid (RA), LDN193189 (BMP inhibitor), SANT1 (inhibitor of hedgehog signaling inhibitor), PdBU (protein kinase C activator) and Y-27632 by turning on the transcription factor PDX1. By the end of S3D2, >80% of the cells should be induced to express PDX1 (Figure 3C).

### 3.6 Pancreatic endoderm (stage 4, steps 24–26)

The pancreatic endoderm stage is induced in S4 medium for the next 3 days with same factors as S3 medium, exception of the innovative combination of FGF2 with FGF7 and drastically lower RA concentration. This stage aims to optimize PDX1+ cells. FGF2/7 synergically increase the HHEX expression whereas repress the beta-cell transcription factor NKX6.1 expression in the following stages. The end of this stage is an important quality-control checkpoint, where should generate >30% PDX1+/NKX6.1 + cells (Figure 3D) to ensure the ultimate expression of SST at the terminal of the SC-delta cell differentiation.

### 3.7 Endocrine precursors (stage 5, steps 27–29)

In stage 5, cells are induced to endocrine precursors with continuous FGF2/7 treatment to further increase SST and HHEX expression, XXI (γ-secretase inhibitor) to downregulate Notch signaling pathway, Betacellulin (member of epidermal growth factor), ALK5 inhibitor II, RA, SANT-1, LDN193189, Zinc Sulfate and heparin. Distinct with the methodology of beta-cell differentiation, thyroid hormone (T3) is depleted since stage 5, which has been reported to repress the expression of SST (Rezania et al., 2014).

### 3.8 Immature SC-delta cells (stage 6, steps 30–36)

To optimize the derivation of delta cells, at the beginning of this stage, the 2D-plated cells are dissociated into single cells by Accutase, and suspended with S6 medium in low-attachment 6-well plates at a density of 6 × 10^6^ cells/well for 3D cultivation (Figure 1B). The medium in this stage contains XXI, LDN193189, Zinc Sulfate, heparin, N-acetyl cysteine (NAC, promotes the proliferation), and R428 (AXL inhibitor) for 5 days of culture. 24 h after suspension, cells begin to aggregate to islet-like organoids.

### 3.9 SC-delta cell maturation and assessment (stage 7, steps 37–89)

The induced SC-delta cells in 3D culture need time to be mature, thus we develop the S7 medium with combination of Zinc Sulfate, heparin and α-Tocopherol (Vitamin E). After S7D6, >20% of SST+/CHGA+ and >25% SST+/PDX1 + cells are generated, which can be statistically analyzed by flow cytometry (Figures 3E, F) and ICC (Figures 3G, H).

## 4 Step by step procedures

This directed delta cell differentiation protocol takes about 4-5 weeks to complete, excluding 1-2 weeks to expand stem cells prior to seeding (Figure 1). The protocol has no pause points until the end of differentiation, when 1-2 more weeks may be required to assess the SC- delta cells. Therefore, given the timing of stem cell expansion, it is recommended that differentiation starts every week or so to ensure that the supply of cells is continuous, thus minimizing the time lag. As mentioned above, quality control at the end of early stages can usually help to check differentiation efficiency by flow cytometry, ICC and qRT-PCR.

### 4.1 Stage 0: stem cell culture and seeding for differentiation • duration 7 days

1. Prepare 6-well plates for cell culture by coating with at least 700uL/well of diluted Matrigel for 2 h at 37°C. DMEM/F12 is used for dilution with the ratio described in the Matrigel product information. After coating, aspirate the DMEM/F12 and the plates are ready for use.
2. Thaw a vial of hPSCs in a water bath at 37°C. Mix thoroughly with 5 mL of mTeSR1 (warm at RT before use) with 10 µM Y-27632. Centrifuge cells at 300 g for 5 min, at RT.
3. Aspirate the supernatant medium and resuspend the cells in mTeSR1 containing 10 µM Y-27632.
4. Count cells and seed into Matrigel treated six-well plates at a minimum cell density of 0.2 × 10^5^ cells/cm^2^.
5. Incubate plates at 37°C in 5% CO_2_. From the second day, feed stem cells with 2 mL/well of mTeSR1 (without Y-27632) every day. Once the cells have reached 80%–90% confluency, they can be passaged or seeded for differentiation. The typical time frame for this process is 5–7 days, with variations contingent upon the growth rate of the individual cells.

Caution: Do not start differentiation directly after thawing, but allowing at least one passage before seeding for differentiation, since cells need time to recover after thawing from the cryopreservation.

Critical Step: It is important to ascertain that the morphology and proliferation rate of the cells are consistent with those observed prior to cryopreservation. We recommend using qRT-PCR or immunostaining to check pluripotency markers such as OCT4 and NANOG.

Critical Step: It is recommended that an adequate number of cells be propagated to ensure the maintenance of a passage and differentiation. Therefore, it is advised that a propagation plan be developed for each experiment.

a) For cell passage and propagation:

6. Before passing the hPSCs, prepare a new Matrigel-coated 6-well plate as described in step 1 to continue the propagation.
7. Aspirate the mTeSR and dissociate cells with 1 mL of gentle cell dissociation reagent per well. Incubate at RT for 3 min.
8. When the edge of cells begins to be clear, remove gentle cell dissociation reagent and add 1 mL of mTeSR1. Scrape off cell colonies using a cell scraper, gently pipette cells up and down to mix well with mTeSR1 and count the cells.
9. Add fresh mTeSR1 to a new Matrigel-coated 6-well plate and seed the cells at a density of at least 0.2 × 10^5^ cells/cm^2^.

b) For differentiation:

10. Prepare Matrigel coated 24-well plates before seeding.
11. Aspirate the mTeSR1, rinse the cells once with PBS, add 1 mL TrypLE per well to dissociate cells into single cells. Place cells at 37°C for 5 min, after which at least 95% of the cells will be dissociated. Add 1 mL of mTeR1 per well and gently pipette up and down to stop dispersing.
12. Centrifuge at 300 g for 5 min, discard supernatant and resuspend the cells in mTeSR1 with 10 μM Y-27632.
13. Count cells and seed into pre-treated 24-well plates at a density of approximately 1.25 × 10^5^ cells/cm^2^.

Caution: Cell colonies rather single cells during passage can properly avoid karyotypic abnormalities, due to excessive passaging of stem cells. We also remind you to initially expand and cryofrozen hPSCs to build a cell bank which provides new **cells** after long passages.

### 4.2 Stage 1: definitive endoderm differentiation • duration 3 days

14. 48 h after seeding for differentiation, cells should reach 100% confluence and form a monolayer (Figure 2). Add Activin A (100 ng/mL) and CHIR99021 (3 µM) to pre-warmed S1 basal medium to prepare S1D1 medium. Table 2 shows the composition of the basal medium for the different steps, and Table 3 summarizes the stored and diluted concentrations of the small molecules used in the different stages. Table 1 will facilitate the calculation of the amount of medium required for each feed throughout the differentiation process.

Caution: If the cells are not 100% confluence after 2 days, extending the incubation time or directly initiation of differentiation would affect the differentiation efficiency. It is essential to ascertain whether any issues have arisen during the plate seeding phase. Potential complications may include inaccurate cell counting or excessive manipulation, which could compromise cell viability.

Critical Step: To achieve optimum efficiency, differentiation factors must be added freshly before each day’s feeding throughout the entirety of the protocol. It is important to note that there is a two-cycle limit for freeze-thaw cycles. Furthermore, it is advisable to avoid maintaining the medium containing differentiation factors in a water bath for an extended period.

15. Rinse cells once with PBS and add S1D1 medium.
16. After 24 h, replace the medium with S1D2 medium, which containing activin A and a very low concentration of CHIR.
17. On the third day, aspirate the S1D2 medium, gently wash the cells with PBS and add S1D3 medium.

Caution: It is not uncommon for some cell death to occur during stage 1.

Caution: It is important to note that when replacing the medium, the cells should be washed to remove any residual CHIR from the previous medium. Otherwise, the differentiation efficiency may be adversely affected.

Critical Step: Effective endoderm induction at the end of stage 1 is important to the success of the protocol. It is therefore beneficial to check the proportion of FOXA2+ and SOX17+ cells at the end of this stage through ICC (Figure 3A) to ensure that >90% of cells express these markers.

### 4.3 Stage 2: primitive gut tube generation • duration 2 days

18. Prepare S2 medium by adding FGF7 (50 ng/mL) to pre-warmed S2 basal medium.
19. Remove S1D3 medium, gently wash cells once with DPBS, and add S2 medium.
20. Replace the medium with fresh S2 medium after 24 h.

Critical Step: The percentage of FOXA2+ and SOX17+ cells at the completion of this stage can be checked by ICC (Figure 3B). We suggest that typically >85% expression is advantageous for the induction of delta cells.

Caution: On the first day of a new stage, wash the cells with PBS prior to the addition of the new period medium. This procedure will ensure the complete removal of differentiation factors from the previous medium, thus facilitating the differentiation efficiency.

### 4.4 Stage 3: posterior foregut generation • duration 2 days

21. At the beginning of this stage, as some cells congregate while the majority remain in a monolayer configuration, they typically show a “valley-like” morphology (Figure 2). Prepare S3 medium by adding FGF7 (50 ng/mL), RA (1 μM), SANT-1 (0.25 μM), LDN193189 (100 nM), PdBU (500 nM) and Y27632 (10 μM) to the S3 basal medium to obtain the S3 medium and warm in a 37°C water bath before use.
22. Aspirate S2 medium, gently wash cells once with PBS, and add S3 medium.
23. Incubate the cells for 2 days, replace the medium after the first 24 h.

Critical step: Pancreatic transcription factor PDX1 is turned on at stage 3, and should be quantified at the completion of this stage by ICC (Figure 3C) to ensure that >80% of the cells express PDX1.

### 4.5 Stage 4: pancreatic endoderm generation • duration 3 days

24. At the beginning of this stage, although some cell clusters from the previous stage may persist, most cells should exhibit a confluent monolayer (Figure 2). Prepare S4 medium by adding FGF7 (50 ng/mL), FGF2 (20 ng/mL), RA (0.1 μM), LDN193189 (100 nM), SANT-1 (0.25 μM), PdBU (100 nM), Y27632 (10 μM) and ALK5i II (10 μM) to the S4 basal medium and warm in a 37°C water bath before use.

Caution: S4 medium is almost the same as S3 medium, except for the addition of FGF2 and a much lower concentration of RA.

Critical Step: The addition of a combination of FGF2 and FGF7 from S4 onwards will significantly promote the expression of HHEX and SST, which represents a key aspect of this protocol for the induction of delta cells.

25. Aspirate S3 medium, gently rinse cells once with PBS and add S4 medium.
26. Culture the cells for 3 days, changing the medium every day.

Critical Step: It is recommended to perform quality control at this stage by checking the percentage of PDX1+ and NKX6-1+ cells by ICC (Figure 3D). At the end of this stage, cells expressing both PDX1 and NKX6-1 should be >30%.

### 4.6 Stage 5: endocrine precursors induction • duration 3 days

27. At the beginning of this stage, the cells should still be in a monolayer, often with some clusters and sometimes small holes (Figure 2). Prepare S5 medium by adding FGF7 (50 ng/mL), FGF2 (20 ng/mL), RA (0.05 μM), LDN193189 (100 nM), SANT-1 (0.25 μM), Zinc Sulfate (10 μM), XXI (1 μM), ALK5i II (10 μM), Betacellulin (20 ng/mL) and heparin (10 μg/mL) to the S5 basal medium and warm in a 37°C water bath before use.

Critical Step: During stage 5, pancreatic progenitor cells convert into endocrine cells. The continuous addition of FGF2 and FGF7 to the S5 medium is crucial for effective induction of delta cells.

28. Aspirate the S4 medium, gently rinse the cells once with PBS and add S5 medium.
29. Culture the cells with S5 medium for 3 days, replace medium every day.

### 4.7 Stage 6: generating SC-delta cells • duration 5 days

30. Prepare the S6 medium by adding LDN193189 (100 nM), Zinc Sulfate (10 μM), XXI (0.1 μM), R428 (2 μM), NAC (1 mM) and heparin (10 μg/mL) to the S6 basal medium and warm in a 37°C water bath.
31. Before starting this phase, aspirate S5 medium, wash cells once with PBS and add 0.5 mL Accutase per well. Incubate for 5 min at 37°C.
32. When the cells begin to detach from the plate, add 1 mL of S6 medium per well, gently pipette them up and down to complete the dispersion. Mix well and count the cells.
33. Centrifuge at 300 *g* for 5 min to allow the cells to pellet, aspirate the supernatant and resuspend the cells in S6 medium with 10 µM Y-27632.
34. Pipette 6 × 10^6^ cells per well into ultra-low attachment 6-well plates, add S6 medium with 10 µM Y-27632 to top up to 3 mL. Place cells on an orbital shaker at 100 rpm (Figure 1B) and culture at 37°C, in 5% CO_2_.
35. After 24 h, when the cells begin to gradually aggregate into spheroid-like clusters, place on an ultra-clean bench tiltly. Wait for 3 min to allow the cells to sink to the bottom, gently aspirate approximately 2 mL of the top medium, and add 2–3 mL of fresh S6 medium.
36. Continue to feed the cells with S6 once a day. Over the next few days, the cell clusters should become bigger and more rounded, eventually form islet-like cell clusters with stable size of approximately 200–250 µm in diameter (Figure 2).

Critical Step: After suspending, cells can be lost up to 50% during aggregation, depending on the differentiation efficiency. Using this method, most endocrine cells tend to aggregate into clusters, while any remaining progenitors and other cell types are susceptible to death during this process. Hence, this aggregation step is prone to purify the endocrine cell population, therefore increasing the proportion of delta cells obtained by induction.

### 4.8 Stage 7: SC-delta cell maturation • duration 14–21 days

37. Prepare S7 medium by adding Zinc Sulfate (10 μM), α-Tocopherol (10 μM), NAC (1 mM) and heparin (10 μg/mL) to the S7 basal medium and warm at 37°C water bath.

Critical Step: We have improved the S5-S7 medium by removing T3, which has been reported to decrease the expression of SST.

38. Aspirate S6 medium with the method described in step 35 and add S7 medium.

Caution: Remove S6 medium as much as possible and wash the cell aggregates once with S7 medium.

39. Culture the cells on the orbital shaker. Feed cells every other day with S7 medium until the end of differentiation when cells are to be assessed.

### 4.9 Intracellular flow cytometry • duration 2 days

40. At the end of the desired stage, aspirate the medium in plates, wash with PBS, and add 0.5 mL TrypLE/well. For aggregated cell spheroids after stage 5, pipette them into a microcentrifuge tube using a P1000 pipettor, wash with PBS, and add 1 mL TrypLE. Incubate at 37°C for 5–10 min.
41. Gently pipette cells, add appropriate volume of corresponding medium to terminate dispersing. Mix well and transfer to a microcentrifuge tube.
42. Centrifuge at 300 g for 5 min at RT.
43. Aspirate the supernatant and add 0.5 mL of 4% (w/v) PFA solution for 15 min at RT.

Caution: We recommend using 4% PFA in a chemical fume hood.

44. Centrifuge at 300 g at RT for 5 min.
45. Dispose the PFA appropriately, resuspend cell pellets in 1 mL PBS and mix well.

Pause point: The fixed cells can be stored at 4°C for up to 2 months.

46. Centrifuge at 300 g for 5 min at RT.
47. Discard supernatant, add 0.5 mL 1× Perm/Wash buffer, mix well and incubate for 20 min at RT
48. Prepare primary antibodies by diluting with 1× Perm/Wash buffer as described in Table 4. Cells can be stained with a variety of markers, depending on the compatibility of antibodies.
49. Centrifuge the sample at 300 g for 5 min.
50. Discard the buffer and resuspend cells in 0.2 mL primary antibody solution. Incubate overnight at 4°C.
51. Prepare the appropriate secondary antibodies by diluting 1:500 in 1× Perm/Wash buffer.
52. Centrifuge the samples at 300 g for 5 min at RT.
53. Remove the buffer with primary antibodies. Resuspend the cells in 0.5 mL 1× Perm/Wash buffer to wash, and centrifuge at 300 g for 5 min at RT.
54. Repeat step 53 to wash one more time.
55. Discard the buffer. Resuspend the cells in 0.2 mL of the prepared secondary antibodies, mix well and incubate for 2 h at RT.
56. Centrifuge at 300 *g* for 5 min at RT. Wash the sample twice with 1× Perm/Wash buffer and resuspend the cells in 0.4 mL 1× Perm/Wash buffer with 2% FBS and 1 mM EDTA.
57. Transfer the stained cell suspension into a flow cytometer polystyrene tube through its filter cap. Run samples on the flow cytometer in 1 day.

**TABLE 4:** Immunostaining antibodies.

Primary antibodies	Dilution	Compatible secondary antibodies
FOXA2	1:400	Anti-rabbit alexa fluor 488 (1:500)
SOX17	1:200	Anti-mouse alexa fluor 546 (1:500)
PDX1	1:100 (for ICC)	Anti-goat alexa fluor 488 (1:500)
	1:100 ((for flow cytometry)	Anti-goat alexa fluor 594 (1:500)
NKX6.1	1:100	Anti-mouse alexa fluor 546 (1:500)
SST	1:100 (for ICC)	Anti-rat alexa fluor 488 (1:500)
SST	1:200 (for flow cytometry)	Anti-mouse alexa fluor 488 (1:500)
CHGA	1:100	Anti-rabbit alexa fluor 647 (1:500)

Critical Step: Stem cells should be stained at the same time with samples, as it is often necessary to use stem cells as a negative biological control when running samples on a flow cytometer, as well as a secondary antibody only control (Figures 3E, F).

### 4.10 Immunocytochemistry (ICC) • duration 2 days

58. At desired stage for detecting, aspirate the medium in plates, wash with PBS, and add 0.5 mL TrypLE/well.
59. Disperse the cells by gently pipette up and down, then add corresponding medium to terminate dispersing. Transfer the solution to a microcentrifuge tube and centrifuge at 300 g for 5 min at RT.
60. Aspirate the supernatant, resuspend the cells with a density of 6.25 × 10^5^ cells/mL in the medium, and drop 0.2 mL of cell solution into Matrigel-coated confocal dishes.
61. After 4 h, check for cell attachment, aspirate medium and wash with PBS.
62. Aspirate PBS and add 0.2 mL 4% PFA to cover the cells. Incubate at RT for 15 min.
63. Dispose PFA properly. Rinse the cells with PBS,
64. Add 0.2 mL 0.5% Triton X-100 in PBS to permeabilize cells for 20 min at RT.
65. Block the sample with 0.2 mL 0.1% Triton X-100 in PBS with 5% donkey serum for 30 min at RT.
66. Prepare primary antibodies by diluting in primary antibody dilution buffer as described in Table 4.
67. Remove blocking solution, add diluted primary antibodies and incubate overnight at 4°C.
68. Prepare the appropriate secondary antibodies by diluting 1:500 in secondary antibody dilution buffer.
69. Remove the buffer containing primary antibodies, wash 3 times with PBS, and add the secondary antibodies.
70. Incubate for 2 h at RT, avoiding exposure to light.
71. Discard secondary antibodies, wash 3 times with PBS solution and add 1:1000 DAPI in the same buffer for nuclear stain. Incubate for 15 min at RT avoiding light exposure.
72. Remove DAPI nuclear stain and wash 3 times with PBS solution.
73. Store confocal dishes in PBS at 4°C prior to imaging by confocal or widefield fluorescence microscopy.

### 4.11 Histology for aggregated clusters • duration 2 days

74. To immunostain S6 or S7 aggregated spheroids, first, remove the cell clusters into a microcentrifuge tube using a P1000 pipettor. Wait for a few minutes until cell clusters settle down, then remove the medium from the top. Add 1 mL PBS and gently invert the centrifuge tube to clean the cell clusters.
75. When cell clusters settle down, remove the PBS, add 0.5 mL of 4% PFA solution and place at RT for 1 h.
76. Discard the PFA and rinse three times with PBS.
77. Remove PBS and suspend the cell pellets in 0.5 mL of 30% sucrose solution for dehydration at 4°C overnight.
78. Carefully remove the sample from the microcentrifuge tube using a P200 pipettor and embed organoids in OCT.
79. Snap-freeze the samples in liquid nitrogen, and store at −80°C.
80. Cut sections at 10 μm using a microtome and section on glass slides using standard histology techniques.

Pause Point: Sectioned slides can be stored at room temperature for at least 1 year.

81. Circle the location of cell clusters with an immunohistochemistry pen prior to staining.
82. Rinse the glass slides 3 times with PBS.
83. For immunostaining, please see step 64–72.
84. Aspirate PBS and apply two drops of antifade reagent, then place a coverslip on top and seal the slides with clear nail varnish.

Caution: Be careful to avoid air bubbles when place the coverslip, especially where the organoids locate.

85. Store samples in a slide box at 4°C before imaging with a confocal or widefield fluorescence microscope.

### 4.12 qRT-PCR • duration 7 h

86. Aspirate the medium in the plates needed to be detected, wash with PBS, and add 0.5 mL TrypLE/well. For aggregated cell clusters, pipette them into a microcentrifuge tube using a P1000 pipettor, wash with PBS, and add 1 mL TrypLE. Incubate at 37°C for 5–10 min.
87. Add 1 mL of the medium and mix well, transfer the cell suspension to a microcentrifuge tube and centrifuge at 300 *g* for 5 min at RT.
88. Aspirate the supernatant and add 600 μL of RLT (with 1:100 Methyl-β-cyclodextrin) to lyse the cells.

Pause Point: Lysed cells can be stored in RLT buffer at −80°C for several months.

89. According to the manufacturer’s instructions, proceed mRNA extraction, reverse transcription, as well as PCR reactions. Sequences of primers are listed in Table 5.

**TABLE 5:** qRT-PCR primers.

Marker	Forward primer (5′-3′)	Reverse primer (5′-3′)
*GCG*	AGC​TGC​CTT​GTA​CCA​GCA​TT	GAG​ATT​TCC​CAG​AAG​AGG​TCG
*INS*	AGG​CCA​TCA​AGC​AGA​TCA​CT	GCA​GCC​TTT​GTG​AAC​CAA​CAC
*SST*	TGG​GTT​CAG​ACA​GCA​GCT​C	CGCTGTCCATCGTCCTG
*PDX1*	CGT​CCA​GCT​GCC​TTT​CCC​AT	CGG​AAC​TTT​CTA​TTT​AGG​ATG​TGG
*HHEX*	CGA​GAC​GCA​GAA​ATA​TCT​CTC​T	CGA​TTC​TGA​AAC​CAG​GTT​TTG​A
*CHGA*	CGC​AAA​CCG​CAG​ACC​AGA​GGA	AGC​TCT​GCT​TCA​ATG​GCC​GAC​A
*NKX6.1*	GGG​CTC​GTT​TGG​CCT​ATT​CGT​T	CCA​CTT​GGT​CCG​GCG​GTT​CT
*CHE1*	CGT​GCT​CAA​CAA​TGT​CGA​TTC​TG	GTC​CAT​CAT​GTA​ATT​GTT​CCA​GCG
*ESE3B*	ATC​AGA​GGC​AGT​GGC​TCA​GCT​A	ACC​AGT​CTT​CGT​CCA​TCC​ACA​C
*ETV1*	GCA​AGA​AGG​CTT​CCT​GGC​TCA​T	CCT​TCC​CGA​TAC​ATT​CCT​GGC​T
*GABRG2*	GCA​CAC​TCA​TTG​TCG​TCC​TAT​CC	CAA​TGG​TGC​TGA​GGG​TGG​TCA​T
*ISL1*	GCA​GAG​TGA​CAT​AGA​TCA​GCC​TG	GCC​TCA​ATA​GGA​CTG​GCT​ACC​A
*LCORL*	TAT​GGA​CCA​CGG​CTA​CGA​AGA​G	TGA​CTG​TGA​AGA​ATC​AAG​AGA​TGG
*LEDGF*	AGG​CAG​GAG​TAG​TGA​CAA​CAG​C	CTC​TCT​GAA​GGA​CAG​GGC​TGT​T
*NEC1*	CCA​GAT​GTG​CAG​GAG​AAA​TTG​CC	CCG​TCA​CAA​TGC​CAT​CCA​GCA​T
*PDLIM4*	TGA​TGA​CAG​CAA​GGC​TCA​GGC​A	AGG​CTT​GGT​CTG​CCA​TCT​TCT​G
*PRG4*	TGT​GAC​TGC​GAC​GCC​CAA​TGT​A	GGT​TTG​AGA​TGC​TCC​TGA​AGG​TG
*RGS2*	CTC​TAC​TCC​TGG​GAA​GCC​CAA​A	TTG​CTG​GCT​AGC​AGC​TCG​TCA​A
*SFRP3*	GCT​ACA​CAG​AAG​ACC​TAT​TTC​CG	CCG​TGG​AAT​GTT​TAC​CAG​AGA​GG
*SHARP1*	CTG​GGA​CAT​CTG​GAG​AAA​GCT​G	AGT​GGA​ACG​CAT​CCA​AGT​CGG​A
*POU3F*	GTG​TTC​TCG​CAG​ACC​ACC​ATC​T	CGC​GAT​CTT​GTC​CAG​GTT​GGT​G
*GAPDH*	TGC​ACC​ACC​AAC​TGC​TTA​GC	GGC​ATG​GAC​TGT​GGT​CAT​GAG

### 4.13 Timing

The protocol takes approximately 4-5 weeks to induce the differentiation of delta cells, plus 1-2 weeks for stem cell expansion and differentiation efficiency evaluation. The time needed for culturing cells each day will depend on plate configurations.

### 4.14 Production of SC-delta cells

Steps 1–13: stem cell culture and seeding differentiations (stage 0), 7 days (0.5–2 h/d hands-on)
Steps 14–17: definitive endoderm differentiation (stage 1), 3 days (0.5–1 h/d hands-on)
Steps 18–20: primitive gut tube generation (stage 2), 2 days (0.5–1 h/d hands-on)
Steps 21–23: posterior foregut generation (stage 3), 2 days (0.5–1 h/d hands-on)
Steps 24–26: pancreatic endoderm generation (stage 4), 3 days (0.5–1 h/d hands-on)
Steps 27–29: endocrine precursors induction (stage 5), 3 days (0.5–1 h/d hands-on)
Steps 30–36: generating SC-delta cells (stage 6), 5 days (0.5–1.5 h/d hands-on)
Steps 37–39: SC-delta cell maturation (stage 7), 14–21 days (0.5–1 h/d hands-on)
Steps 40–57: intracellular flow cytometry, 2 days (day 1 with 3 h sample preparation and primary antibody staining, day 2 with 3 h sample preparation and secondary antibodies)
Steps 58–73: immunostaining (ICC), 2 days (day 1 with 3 h sample preparation and primary antibody staining, day 2 with 3 h sample preparation and secondary antibodies)
Steps 74–85: histology for stage 6 aggregated clusters, 2 days (day 1 with 8 h sample preparation and primary antibody staining on day 1, day 2 with 3 h sample preparation and secondary staining on day 2)
Steps 86–89: qRT-PCR, 7 h (RNA extraction 1.5 h, reverse transcription 2.5 h, plate setup and PCR reaction 3 h)

## 5 Anticipated results

**FIGURE 4:**
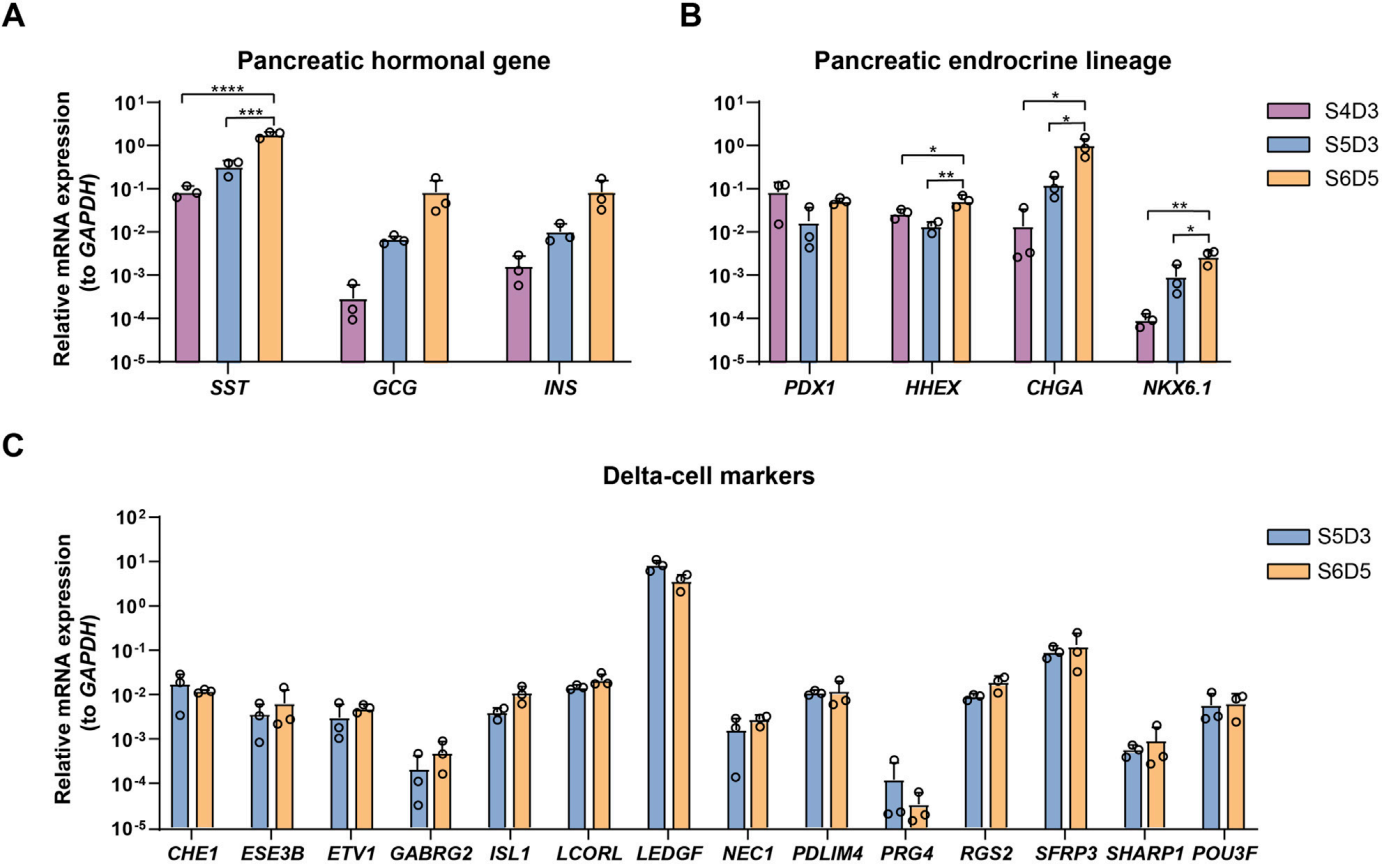
Characterization of SC-delta cells. **(A)** qRT-PCR of pancreatic hormonal gene *SST*, *INS* and *GCG* expression at the end of stage 4, 5 and 6 (n = 3). The ΔΔCt method was used to calculate relative mRNA expression normalized to *GAPDH*. *** p < 0.001; ****p < 0.0001 (one-way ANOVA followed by Tukey’s multiple comparisons test). **(B)** qRT-PCR of endocrine precursor markers *PDX1*, *HHEX*, *CHGA* and *NKX6.1* at the completion of stage 4, 5 and 6. *p < 0.05; **p < 0.01 (one-way ANOVA followed by Tukey’s multiple comparisons test). **(C)** Expression levels of typical delta-cell markers at stage 5 and 6. Data shown in this figure are given from H1 cell line. Values are mean ± s.e.m. from 3 independent differentiations.

The proportion of SC-delta cells (SST+) relative to the total number of cells (DAPI+) generated with this protocol should reach a minimum of 20%. Here, we provide representative data to show the expected results. We initially developed and optimized this protocol using the H1 cell line, which yielded 26.7% SST+/PDX1+ cells and 31.6% SST+/CHGA + cells on S7D7 (Figures 3E, F). Immunostaining of cell clusters from the beginning and end of stage 7 shows the expected proportion of SC-delta cells, although the number of SST + cells may increase slightly as the delta cells mature (Figures 3G, H). Furthermore, we demonstrate the characteristics of delta cell-related gene expression from S4 to S6 through qRT-PCR. The typical pancreatic hormonal genes *SST*, *INS* and *GCG*, all demonstrate a progressive increase in expression from the end of S4 to S6 (Figure 4A). The expression of the endocrine precursor markers *CHGA* and *NKX6.1* also show an increasing trend (Figure 4B). Nevertheless, the pancreatic endoderm progenitor marker *PDX1* and the delta cell marker *HHEX* both demonstrate a decline from S4 to S5, followed by a restoration of levels from S5 to S6 (Figure 4B). Other delta cell markers, including *CHE1*, *ESE3B*, *ETV1*, and *ISL1*, are expressed at similar levels in S5 and S6 (Figure 4C).

**FIGURE 5:**
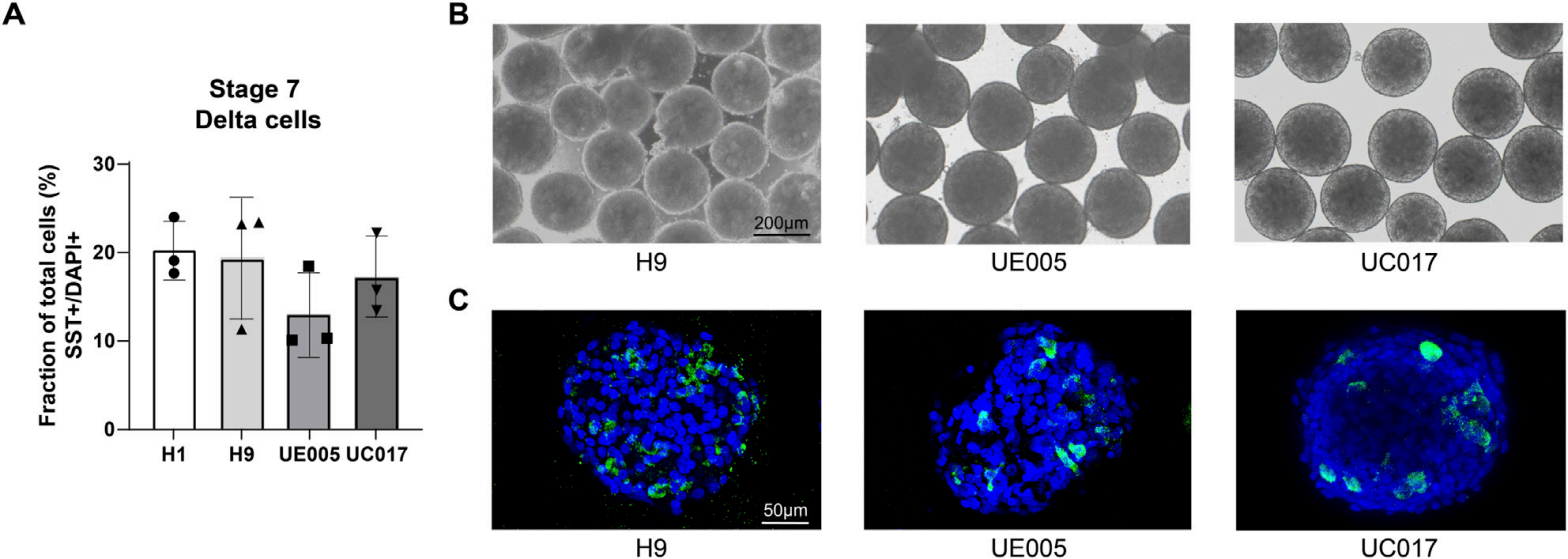
SC-delta cells differentiated from additional stem cell lines. **(A)** Bar graphs representing the proportion of SST + delta cells in stage 7 cell clusters generated from H1 and additional cell lines H9, UE005 and UC017 with this protocol (n = 3 for H1, H9, UE005, and UC017, n represents for independent differentiations). Data are quantitatively counted with immunostaining of histological sections. **(B)** Morphology of stage 7 clusters generated from these three cell lines with this protocol. Scale bar, 200 µm. **(C)** Immunostaining of stage 7 histological sections showing the SST + delta cells generated from three cell lines. Scale bar, 50 µm.

We also induce the delta cell generation following this protocol with other cell lines: H9, UE005 and UC017. All these cell lines are able to form cell clusters after suspension, exhibiting a morphology similar to that of H1 (Figure 5B) and successfully yield SST + delta cells (Figures 5A, C), although the faction of delta cells varies due to differences in differentiation efficiency between cell lines (Figure 5A).

## 6 Discussion

Our primary study reported the directed differentiation of pancreatic delta cells (Chen et al., 2024). Building on this, here we detail a methodology that is accessible, efficient, and amenable. This protocol will provide a stable and sufficient cell source and facilitate future investigations of delta cells, including their physiological roles, drug screening and the construction of islet-like organoids. Undoubtedly, it will advance mechanistic studies, drug discovery and cell replacement therapy in the field of diabetes.

Future work should focus on several other aspects. First, we note that the yields of SC-delta cells from different cell lines are different. Therefore, it is important to perform quality control from the early stages of differentiation. Furthermore, it’s valuable to use additional cell lines based on this benchmark for further comparison. Second, the induced SC-delta cells are less mature than human islet-derived delta cells (Chen et al., 2024). Consequently, future research should concentrate on elucidating the signaling pathways involved in delta cell maturation with a view to optimizing the differentiation method. Third, the identity of additional cell types, beyond those that secrete hormones, remains uncertain. Consequently, elucidating the role of these cells in delta cell generation and their potential as a source for SC-delta induction will facilitate further optimization of this process. Fourth, the establishment of this protocol provides researchers with the ability to explore surface markers for delta cell purification. It is our contention that this methodology will prove invaluable in future studies of delta cell function and the development of novel therapeutic strategies for diabetes.

## Data Availability

The raw data supporting the conclusions of this article will be made available by the authors, without undue reservation.
